# SMS versus voice messaging to deliver MNCH communication in rural Malawi: assessment of delivery success and user experience

**DOI:** 10.9745/GHSP-D-13-00155

**Published:** 2014-01-28

**Authors:** Jessica Crawford, Erin Larsen-Cooper, Zachariah Jezman, Stacey C Cunningham, Emily Bancroft

**Affiliations:** a VillageReach, Balaka, Malawi; b VillageReach, Seattle, WA, USA; c VillageReach, Seattle, WA, USA. Now with Ipas, Chapel Hill, NC, USA.

## Abstract

**Objective:**

To determine the difference in delivery success of health messages delivered through pushed SMS, pushed voice messages sent to personal phones, and voice messages retrieved from a community phone (“retrieved voice messaging”), as well as the difference in quality of the user experience.

**Methods:**

We analyzed the project's electronic monitoring data between September 2011 and June 2013, including demographics, enrollment data, and messages sent and successfully delivered. We also collected and analyzed information from quarterly phone-based surveys with users to assess quality of the user experience, including acceptability, comprehension, new information learned, and reported behavior change.

**Results:**

More than half of subscribers enrolled in the retrieved voice messaging service while nearly one-third enrolled in the pushed SMS service and less than 10% in pushed voice messaging. Message delivery success was highest among pushed SMS subscribers and lowest among retrieved voice subscribers. Overall, 99% of survey respondents reported trusting messages they received, and about 75% of respondents recalled the last message they received and learned something new. Almost 75% of respondents reported that they had already changed or intended to change their behavior based on received messages. Intended or actual behavior change was significantly higher among pushed SMS enrollees than among pushed or retrieved voice messaging enrollees (*P* = .01).

**Conclusion:**

All message modalities led to high levels of satisfaction, comprehension, and new information learned. Due to lower cost, higher delivery success, and higher levels of intended or actual behavior change, SMS is the preferred delivery modality. However, the majority of users included in this study did not have access to a personal phone, and retrieved voice messages provided an opportunity to access a population that otherwise could not be served. Providing multiple methods by which users could access the service was crucial in extending reach beyond literate personal phone owners.

## Abstract (precis)

Mobile SMS health messages had higher successful delivery and led to higher intended or actual behavior change among subscribers than voice messages. Providing multiple delivery modalities led to greater overall access.

## BACKGROUND

Despite significant progress toward improving maternal and child health, Malawi still has high maternal and infant mortality; the maternal mortality ratio is 675 maternal deaths per 100,000 live births and the under-5 mortality rate is 112 deaths per 1,000 births.^1^ Underlying causes of poor health for women and children include limited availability of timely and reliable health information for decision-making and poor access to and use of health facilities.^2^ Knowing when and where to go for care is integral to maximizing health care access and use and to reducing maternal and child mortality.

With increased availability and use of mobile technology, mHealth is becoming a widely used strategy to address barriers to accessing health information and care.^3^^–^^5^ mHealth is the use of mobile phones to promote healthy behavior, increase use of health services, improve adherence to health advice, and increase access to health information. One growing application of mHealth is mobile messaging, whereby health information and promotion messages are sent directly to clients. Previous studies have highlighted ways in which mobile messaging can be successful in smoking cessation, weight loss, diet and physical activity, treatment adherence, and disease management.^6^^,^^7^

Mobile health messaging has been successful in public health programs, such as for smoking cessation and weight loss.

Mobile messaging is increasingly being applied to improve reproductive, maternal, neonatal, and child health (RMNCH), and there is growing evidence of its effectiveness.^8^^–^^11^ For example, a U.S.-based program, text4baby, sent text messages to traditionally underserved pregnant women encouraging them to adopt healthy attitudes, beliefs, and behaviors during pregnancy. The program was effective in increasing agreement with the statement, “I am prepared to be a new mother.” However, it did not lead to significant behavior change among enrollees.^10^ The effects of the intervention were greater among enrollees with more education, suggesting that literacy and comprehension of messages may play crucial roles in the effectiveness of mHealth projects.

Literacy may be a critical factor in the effectiveness of mHealth projects.

An mHealth project in Zanzibar called Wired Mothers sent pregnant women text messages with appointment reminders and health education information, and it also gave enrollees phone vouchers to call health care providers directly with questions. The project significantly increased facility-based births among urban women but had no significant effect on rural women.^11^

In Thailand, the “Better Border and Healthcare Program” found that sending users text messages regarding antenatal care visits and immunizations increased the number of mothers and children attending scheduled appointments.^12^

Mobile messages are generally delivered by either short message service (SMS) or voice messaging; both offer distinct advantages and disadvantages. SMS is available on an estimated 98% of mobile phones, does not require technical expertise to use, and is adaptable to multiple mHealth purposes.^13^ SMS messages can be accessed at user convenience and can be delivered to phones that are turned off or have flat batteries. In addition, telecom costs for SMS are generally less expensive than costs for voice communication. Given these advantages, it is not surprising that SMS is the most common delivery method for mobile development services.^13^

Voice messages, although used less often in mHealth projects, offer the advantages of accessibility to illiterate populations, ability to contain more information per message than SMS, and ability to be recorded in any language (not all languages/characters are supported by SMS).^14^ In addition, recorded voice messages allow information to be conveyed by a clinical “character,” as used in Bangladesh by the Mobile Alliance for Maternal Action (MAMA), to build rapport and trust over time.^15^

SMS has several advantages over voice communication, but voice messages may provide accessibility to illiterate populations.

Research comparing the outcomes of voice versus SMS messaging is limited in the mHealth literature, although user preference has been examined in a small number of studies. A U.S.-based study among literate English speakers found that 72% of participants in an mHealth program targeting exercise, diet, and smoking preferred to enroll in automated voice messages over SMS. Those who preferred SMS tended to be younger and to have higher levels of comfort with computers and higher levels of SMS use.^16^

The Mobile Technology for Community Health (MOTECH) initiative in Ghana used a “Mobile Midwife” application to provide pregnant women with health education and reminders to access necessary medical services. Messages could be received in voice or SMS format, depending on the enrollees' preference; 99% of enrollees chose voice.^17^

Questionnaires administered to potential mHealth users in Argentina, however, found that the users were similarly open to receiving SMS and voice messages during pregnancy, with 96% of participants willing to receive SMS messages and 87% willing to receive voice messages.^18^ To our knowledge, no studies have been conducted to determine the difference in acceptability, comprehension, or behavior change between users receiving messages through voice compared with SMS formats.

This article describes the messaging component of an mHealth pilot project in Malawi aimed at improving knowledge and uptake of home- and facility-based MNCH care practices using both SMS and voice message delivery modalities. We report findings from the pilot examining differences between SMS and voice messaging with regard to delivery success and quality of user experience, including acceptability, comprehension, new information learned, and reported behavior change.

## PROJECT DESCRIPTION

*Chipatala Cha Pa Foni* (CCPF) (“Health Center by Phone”) is a mobile health project of Concern Worldwide's Innovations for Maternal, Newborn & Child Health initiative, and its partners VillageReach and the Malawi Ministry of Health (MOH). A nationwide campaign generated more than 6,000 ideas of how to improve quality of health services from community members in Malawi; one of the winning ideas became CCPF.

**Figure:**
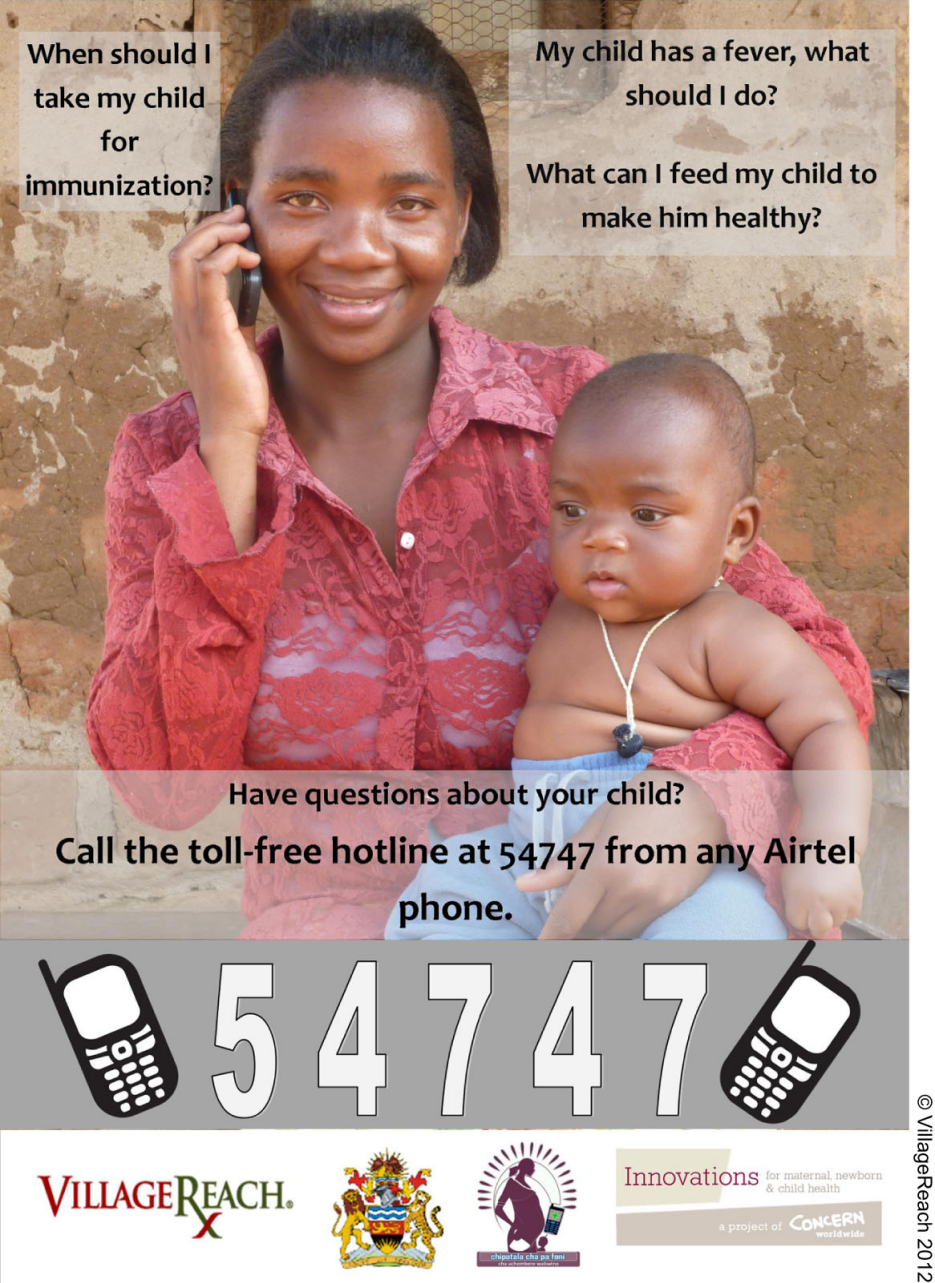
A communication poster encourages caregivers of children to call the *Chipatala Cha Pa Foni* hotline to get answers to their health-related questions.

CCPF consists of a toll-free hotline offering protocol-based health information, advice, and referrals as well as a mobile messaging service offering automated tips and reminders for pregnant women, guardians of young children, and women of childbearing age. Starting in 2011, CCPF was piloted in 4 rural health center catchment areas in Balaka District in Southern Malawi, representing a population of 32,000 women of childbearing age, 24,000 children under 5, and 7,000 expected pregnancies per year. The pilot project aimed to improve the coverage and quality of RMNCH in Balaka District. Early success and interest has resulted in scale up to an additional 3 districts.

The hotline component of CCPF is staffed by individuals trained in RMNCH using modules from the MOH's curriculum for community health workers (CHWs). When answering calls, hotline workers are prompted by software on touch screen devices to identify clients' symptoms and/or information needs. They answer questions, offer health advice, and provide information on when and where to seek care if clients have symptoms or danger signs that cannot be treated safely at home.

**Figure:**
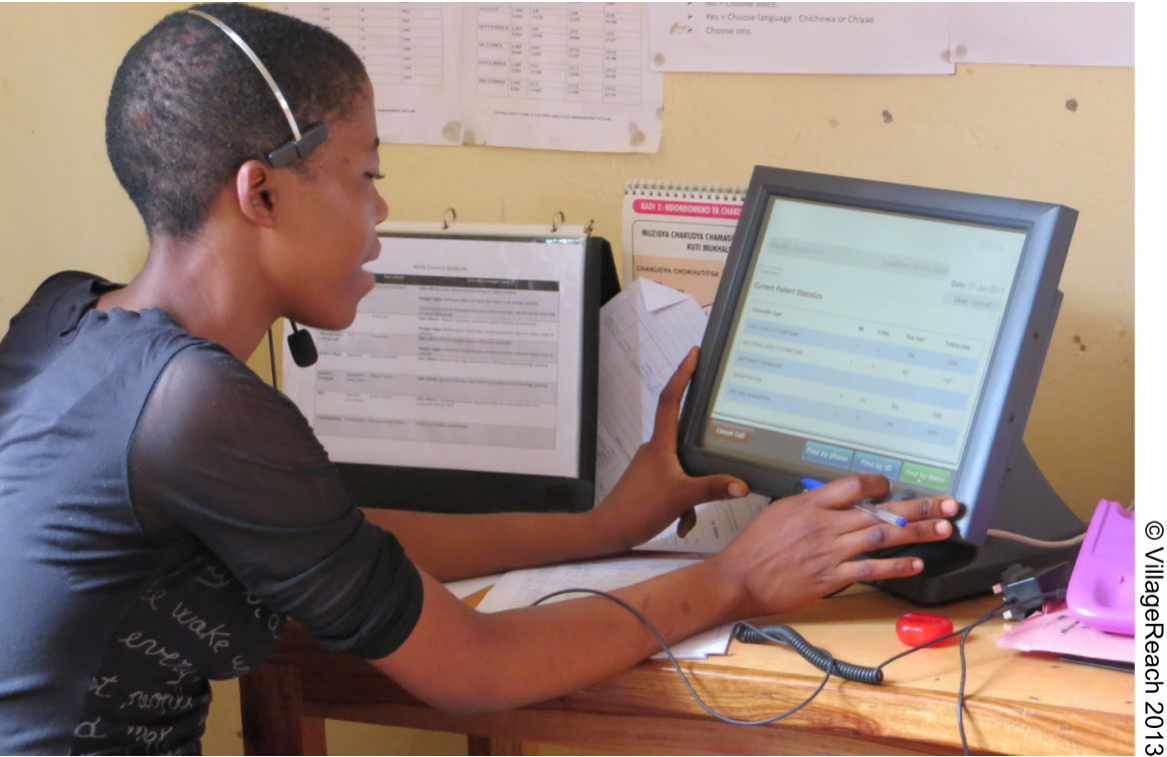
A hotline worker answers an incoming calling using a touch screen device that prompts the worker to identify the client's symptoms and information needs.

Hotline workers also offer eligible clients the opportunity to enroll in the tips and reminders mobile messaging service where they can access voice or SMS messages on a weekly basis. Messages offer timely information and reminders about important health services, based on the estimated date of delivery for pregnant women or the child's age for caregivers of children under 1. The tips and reminders are intended to elicit behavior change by helping clients understand their susceptibility to and seriousness of common maternal and child health illness, and they provide cues to action, reminding pregnant women and caregivers of children to seek timely preventive care. Messages for women of childbearing age were added later in implementation.

During project design, we found that low literacy and phone ownership among the target population may limit access to the service. Although an estimated 75% of women over 15 years old in Balaka District are literate,^1^ poor women and women living in rural areas are less likely to be literate than their wealthier and urban counterparts.^19^ Furthermore, a baseline assessment found that only approximately one-third of women of childbearing age in the catchment area owned a mobile phone.^20^ Thus, we developed 3 message delivery modalities in order to better accommodate individuals with low literacy or without access to personal phones:

1. **Pushed SMS Messages:** Clients with access to a personal or household mobile phone can automatically receive SMS messages on their mobile phones.
2. **Pushed Voice Messages:** Low-literacy clients with access to a personal or household phone can receive weekly recorded voice messages that are sent to the phone at a specific time each week.
3. **Retrieved Voice Messages:** Clients without access to a personal phone can access their weekly messages by calling the toll-free hotline from any phone and entering an appropriate code to hear their message.

Voice messages are more expensive to deliver so clients with personal phones who identified as being literate were generally encouraged to register for SMS. Low-literacy clients were offered the option of registering for voice messages. Prior to launch, each message delivery option was pilot tested among a small group of users in Balaka to ensure delivery and accessibility.

Due to low phone ownership in Balaka, we provided an additional point of access to CCPF. We originally planned to distribute phones to Malawi's paid cadre of CHWs. However, given the large number of potential users without access to a personal phone, community volunteers from each village were instead recruited in consultation with traditional leaders in the area and provided with a low-cost phone. The volunteers were oriented to the program and provided with small incentives throughout the pilot period to encourage their continued participation. In order to provide phones to all volunteers, we sought partnerships that would allow us to stretch the budget to provide phones to a larger number of people than originally anticipated. Our telecom partner agreed to provide a discount on their basic low-cost promotional phones, which were ultimately purchased for the project.

**Figure:**
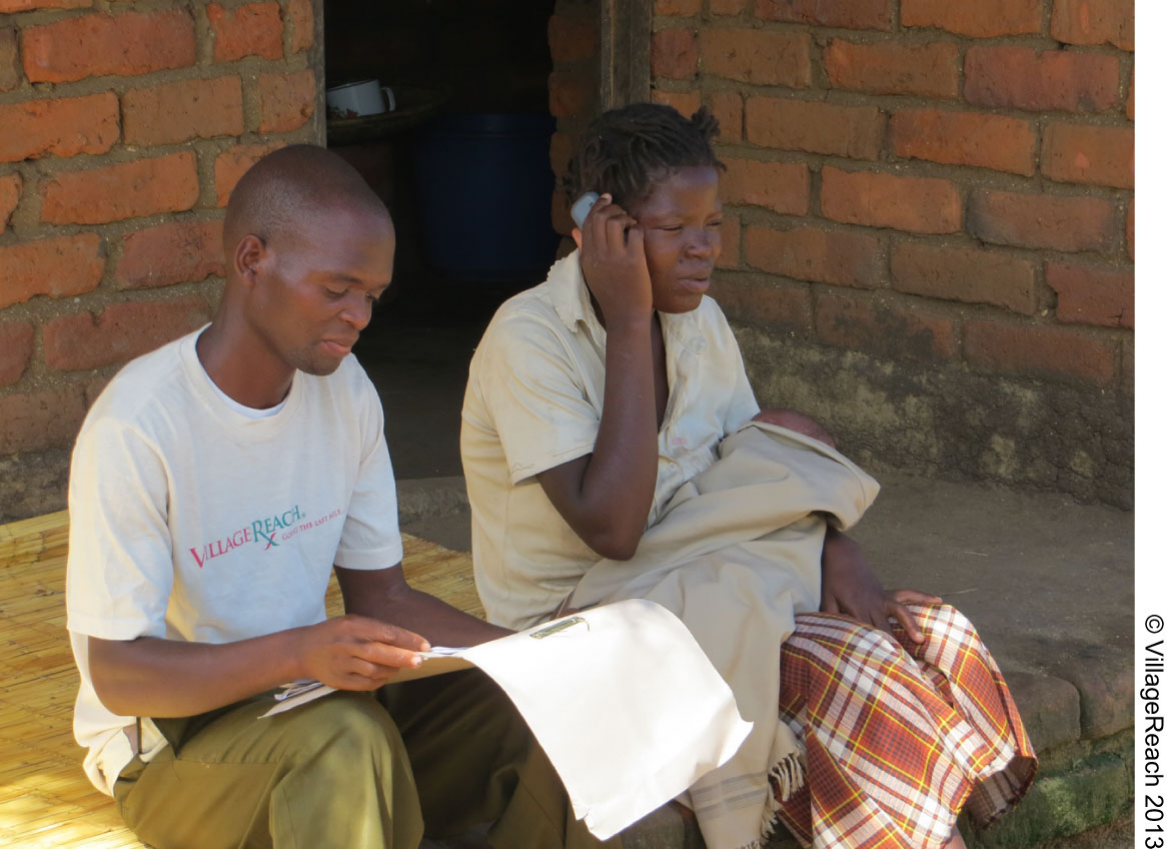
A woman uses a community volunteer's mobile phone to access the health message delivered by the *Chipatala Cha Pa Foni* project.

### Message Development and Content

Messages were developed in collaboration with MOH staff with different areas of expertise, with technical support from PATH, the Grameen Foundation, and BabyCenter. The content was designed to complement national policies and to address local myths and traditions around pregnancy and child rearing. The final content was reviewed and revised by a group of women from Balaka with experience working on RMNCH issues at the community level. Messages were initially developed in English and later translated to Chichewa and Chiyao, the primary local languages spoken in Balaka.

The final content was the same for both voice and SMS messages. However, SMS messages were delivered more frequently than voice messages because SMS messages were shorter than voice messages. Messages were delivered once or twice per week depending on the stage of pregnancy or age of the child. Table 1 provides examples of SMS and voice messages.

SMS messages were delivered more frequently than voice messages because they were shorter than the voice messages.

**TABLE 1.** Sample SMS and Voice Messages

**Subscriber**	**Message Type**	
	**SMS**	**Voice**
**Pregnant Women**	Message 1: When you and your family know that you are pregnant, a visit to ANC will help you understand everything you need to do to keep the baby healthy.Message 2: You and your growing baby need food for energy, body building, and protection. Meat, eggs, greens, vegetables, rice, and fruits help your baby grow.	The ANC is your partner in the pregnancy. It is important to go to all 4 of your visits and to use the tablets that they have given to you. Your baby is continuing to grow! Your baby's fingernails and fuzzy hair are appearing and it's able to do things like swallow and kick. In the next 3 weeks, your baby will double in size. Body building foods like meat, beans and eggs are important for you and your baby. Try to take some every day. And remember; only take medicines given to you by a nurse. Traditional medicines can be dangerous for your baby. You might be feeling that you need to urinate a lot. This is a normal part of pregnancy. Try to drink less water before bed. If you feel pain or burning when you urinate you may have infection.
**Caregivers of Children Under 1**	Message 1: Make sure your baby has its vaccination. In the first week, your baby should get polio vaccine by mouth and the BCG vaccine against TB by injection.Message 2: Keep the cord stump clean and dry. Do not force it to fall off. If there is discharge or redness visit the clinic. Infections can be serious.	Congratulations on being a new mom! This week, make sure your baby has all its vaccinations, including the polio vaccine and the BCG vaccine against tuberculosis. You should start breastfeeding immediately and remember to only give your child breast milk for the first 6 months of his life. Carry your baby close to your breasts because it will help give your baby good physical and emotional conditions and help you to grow close. Keep your baby's cord stump clean and dry and do not force it to fall off. If there is discharge or redness visit the clinic. Infections can be serious. Your baby should be placed on his back for sleeping until he is strong enough to roll over himself.

Abbreviations: ANC, antenatal care; BCG, bacille Calmette-Guérin; SMS, short message service; TB, tuberculosis.

Content was reviewed periodically and updates were made to include new information. For example, new information was added in mid-2012 about the launch of a new pneumococcal vaccine in Malawi.

Originally, CCPF relied on volunteers to advertise the service in the community. Later, CCPF staff started to attend antenatal care clinics to inform pregnant women about CCPF and to help them sign up for the service. In addition, large community events were held to inform community members about CCPF and how to access the service.

## DATA AND METHODS

To understand the difference in delivery success and quality between the 3 different messaging modalities (pushed SMS, pushed voice messages, retrieved voice messages), we collected and analyzed information from routine monthly monitoring data and quarterly phone-based surveys with users.

### Routine Monthly Monitoring

Data from the messaging service were monitored monthly from 3 technology sources built to deliver the service:

- **Hotline Software.** Built by Baobab Health, a Malawian nongovernmental organization providing eHealth solutions, the hotline software is protocol-based and guides hotline workers through each call according to information collected through interactions with the client. The software includes demographic information and tips and reminders enrollment.
- **Notification Application.** Built by VillageReach, the application connects the hotline software to a communications server and pushes voice and SMS messages to enrollees with personal phones. This application records data on messages sent and successfully delivered.
- **INTELLIVR (IVR) Software.** Built by Yo! Uganda, a software company in Uganda, IVR hosts incoming calls and also serves as the outgoing-voice gateway for the tips and reminders service.

Monthly data for all enrolled clients from September 2011 through June 2013 were analyzed to describe the number and characteristics of enrollees by delivery modality as well as delivery success rates.

### Quarterly Phone-Based Surveys

Starting roughly 3 months following the launch of CCPF, periodic phone-based surveys were conducted among a sample of enrollees. Clients enrolled in pushed SMS and voice messaging services were randomly selected from a list of all enrollees and were contacted directly using the phone number for message delivery. Because we did not have phone numbers for clients enrolled in the retrieved voice message service, random selection was not feasible. Instead, we sent community volunteers an SMS asking them to identify a woman in their area who had used the service and to notify hotline staff once they gave the community phone over to the client. At that time, the client was contacted directly for the survey.

For all participants, a hotline worker or volunteer administered the questionnaire, which collected information on frequency of messages received and quality of the experience. Quality of user experience was measured as:

- **Acceptability:** proportion of respondents who indicated they trust the messages
- **Comprehension:** proportion of respondents who could describe the last message they received; the description given by the respondent was then checked in the analysis phase against the messages the respondent should have received
- **New information learned:** proportion of respondents who indicated they learned new information about their pregnancy or child's health and could list topics they deemed to be new information
- **Reported behavior change:** proportion of respondents indicating a health behavior change or intent to change behavior based on the messages received. Behavior change intent was scored according to a series of questions regarding specific behaviors targeted in the messaging. For pregnant women, these behaviors include number of antenatal care visits, food and medicine consumption, and place of delivery. For caregivers of children, these behaviors include medicine and vaccinations for the child, exclusive breastfeeding, and number of postnatal care visits.

The survey was implemented at 3, 6, 12, and 18 months following the launch of the service. Data from 266 questionnaires were entered into an Excel spreadsheet and analyzed using STATA version 10.

## RESULTS

### Routine Monthly Monitoring

During the first 2 years of implementation (through June 2013), more than 5,000 pregnant women and caregivers of children under 1 registered for the tips and reminders service. Client registration data were collected starting in July 2011, when clients could begin registering for the service, while monitoring of delivery success rates started in September 2011 when the messaging service was officially launched.

Table 2 displays the number of registered clients and demographics by content type and delivery method. More than half of clients enrolled in the retrieved voice service, nearly one-third enrolled in the pushed SMS service, and less than 10% enrolled in the pushed voice message service. The mean age of pregnant enrollees was 25, and they registered, on average, 16 weeks before their estimated due date. Caregivers of children under 1 registered, on average, 36 weeks before their child's first birthday.

The majority of subscribers enrolled in the “retrieved voice messaging” service because they did not own a personal phone.

**TABLE 2.** Demographics of Registered Users, by Subscriber Type and Delivery Method (July 2011–June 2013)

	**Pushed SMS**	**Pushed Voice**	**Retrieved Voice**
**Pregnant Women**			
No. of registered users	704	238	1,559
Age, mean, y	25	26	25
Employed in formal sector, %	13	9	8
No. of weeks eligible for messages, mean	16	16	16
**Caregivers of Children Under 1**			
No. of registered users	733	224	1,654
Age of child, mean, mo	4	4	4
Female, %	54	53	55
No. of weeks eligible for messages, mean	36	35	36

Characteristics of clients were similar across service types with the exception of occupation. Pregnant women who registered for pushed SMS messages were more likely to be employed in the formal sector than those who registered for (pushed or retrieved) voice messages. Formal sector includes those who reported their occupation as “business,” “health care worker,” or “teacher.” Informal sector includes those who reported their occupation as “farmer,” “housewife,” “student,” or “other.”

The proportion of messages successfully pushed and retrieved varies by month. For pushed messages (SMS and voice), success rates represent the number of messages successfully received after 3 attempts divided by the number of messages attempted. For retrieved voice messages, success rates represent the number of messages successfully retrieved from the IVR menu divided by the number of messages we estimate should have been retrieved each month. Overall success rates for pushed SMS and voice messages ranged from 54% to 64% and were higher for pushed SMS than for pushed voice messages (Table 3). Success rates for retrieved voice messages were lower, at 27% to 38%.

The SMS service had higher delivery success rates than the pushed or retrieved voice messaging services.

**TABLE 3.** Delivery Success of Messages, by Delivery Method and Subscriber Type (September 2011–June 2013)

	**Pushed SMS**		**Pushed Voice**		**Retrieved Voice**	
**Messages**	**Pregnancy**	**Child**	**Pregnancy**	**Child**	**Pregnancy**	**Child**
No. attempted or expected^a^	19,356	20,363	3,022	2,815	32,054	52,829
No. successfully received or retrieved	11,825	13,053	1,820	1,515	12,257	14,455
Percent success	61%	64%	60%	54%	38%	27%

a For retrieved voice messages, the expected number of messages retrieved is calculated weekly based on the number of current subscribers. For pushed SMS and voice messages, the number of messages attempted was based on actual number of attempts.

**FIGURE 1.**
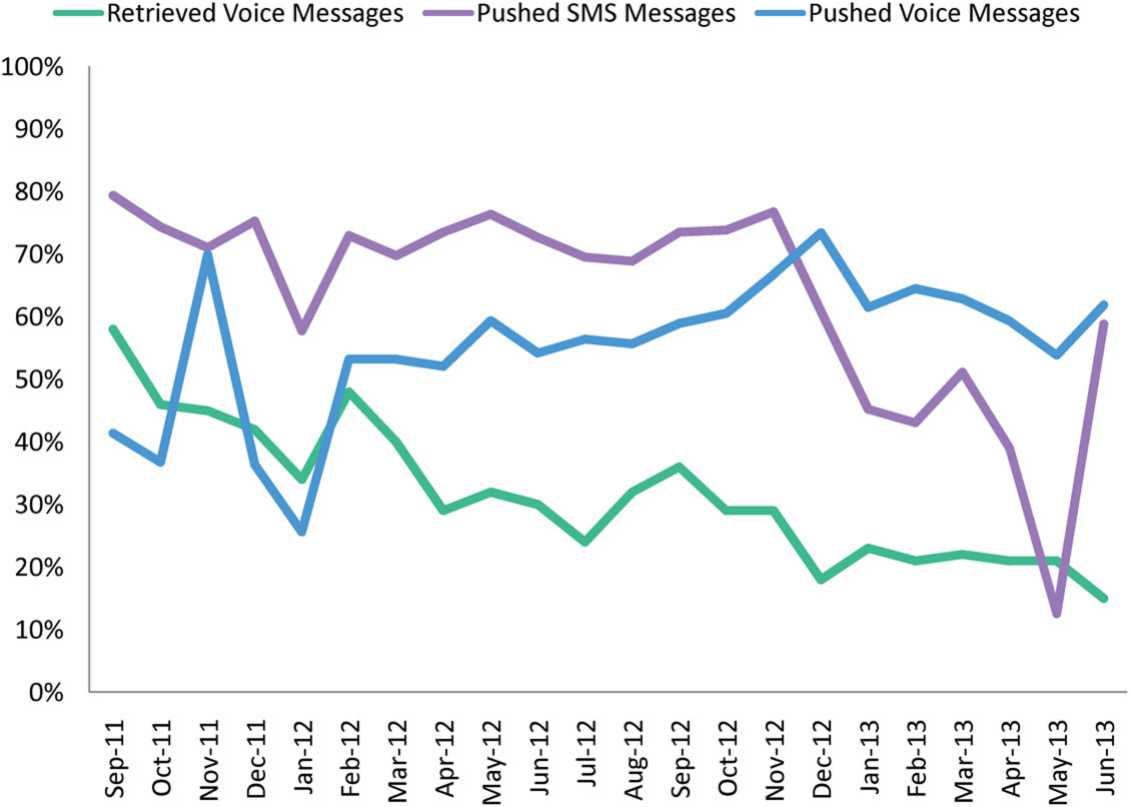
Delivery Success Rates of Messages Over Time, by Delivery Method, September 2011–June 2013 Success rate for pushed SMS and voice messages is the percent of messages successfully received within 3 attempts; for retrieved voice messages, the success rate is the percent of expected messages successfully retrieved.

Delivery success changed over time, with the proportion of pushed voice messages successfully delivered to a personal phone increasing slightly over time, and the proportion of retrieved voice messages successfully delivered decreasing over time (Figure 1). Pushed SMS delivery success rates remained relatively stable over time, with a decline in the beginning of 2013, as a result of a number of technical issues. For example, configuration changes by the telecom provider unintentionally reduced the number of times our system attempted to send messages, resulting in messages being attempted less than 3 times. The issue has since been rectified, but it affected success rates for January–May 2013.

**FIGURE 2.**
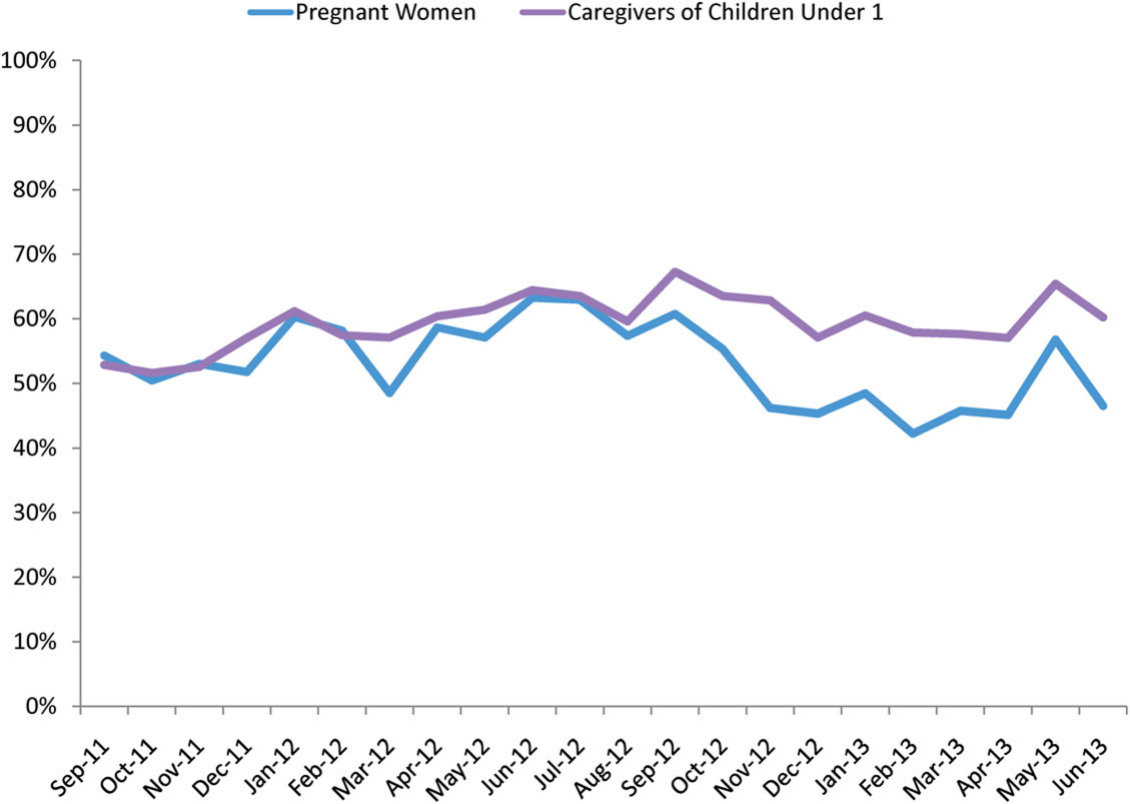
Percentage of Successful Message Retrieval Attempts Among Retrieved Voice Message Subscribers, by Type of Subscriber, September 2011–June 2013

To better understand voice message retrieval rates, we also calculated the proportion of messages successfully played based on the number of attempts (Figure 2). This proportion is calculated from the number of times the message menu is reached (that is, a person calls CCPF's toll-free number and enters “2” to hear a message and then “1” to hear a pregnancy message) and how many times messages are played. Using this definition of delivery success for retrieved voice messages, messages were successfully played in 56% of the instances when the message menu was reached, and success was slightly higher for messages played from the child message menu than messages played from the pregnancy message menu (60% versus 54%).

### Phone-Based Surveys

Hotline workers and volunteers were able to reach 266 total clients for the phone survey. Of those, 96 were enrolled in the pushed SMS service, 30 in the pushed voice service, and 140 in the retrieved voice service (Table 4).

**TABLE 4.** Phone-Based Survey Sample Characteristics, by Delivery Method

	**Pushed**	**Pushed**	**Retrieved**	
	**SMS**	**Voice**	**Voice**	**Total**
	**(n = 96)**	**(n = 30)**	**(n = 140)**	**(N = 266)**
No. of subscribers				
Pregnancy messages	48	13	66	127
Child messages	48	17	74	139
No. of messages received, mean	6.9	3.1	3.7	4.7

Slightly more clients enrolled to receive messages about child health than about pregnancy were included in the survey. At the time of the survey, SMS enrollees reported the highest number of messages received, at an average of 6.9 messages. Pushed voice and retrieved voice enrollees included in the survey had received an average of 3.1 and 3.7 messages, respectively, at the time of survey. Overall, 19% (51) of respondents indicated that they had not yet received any messages, the majority of which (29) were among pushed voice enrollees.

Overall, 99% of respondents reported trusting messages they received (acceptability), and 75% recalled the last message they received (comprehension) (Table 5). Pushed SMS and retrieved voice enrollees were more likely than pushed voice enrollees to recall the last message received, but the observed differences were not statistically significant.

**TABLE 5.** Quality of User Experience, by Delivery Method

	**Pushed**	**Pushed**	**Retrieved**	
	**SMS**	**Voice**	**Voice**	**Total**
**Outcome**	**(n = 96)**	**(n = 30)**	**(n = 140)**	**(N = 266)**
Acceptability	99%	94%	100%	99%
Comprehension	75%	63%	76%	75%
New information	79%	76%	71%	77%
Behavior change (intended or actual)	91%	56%	66%	74%

Missing information was excluded from the analysis. If respondents answered that they had not received any messages, they were not asked questions about the message service.

Just over three-quarters of enrollees indicated that they had learned something new from the messaging service and were able to describe the new information. Pushed SMS enrollees were more likely to respond that they had learned something new.

Almost three-quarters of respondents reported that they had already changed or intended to change their behavior based on the messages they received. Pushed SMS enrollees were significantly more likely to report intended or actual behavior change than voice enrollees (*P* = .01). See box for examples of behaviors that respondents said they intended to change.

SMS subscribers were significantly more likely to report intended or actual behavior change than pushed or retrieved voice messaging subscribers.

**BOX. Quality of User Experience: Selected Examples Reported by Survey Respondents**

**Recalled messages:**

- I should breastfeed my child up to 6 months without giving supplementary foods.
- If the child has shown signs of drying in the mouth, I should run to the hospital.

**New information:**

- I have learnt how to solve small problems without going to hospital.
- The child is supposed to receive measles vaccine.

**Intended or actual behavior change themes:**

- The number of antenatal care visits I plan to attend.
  The types of foods I am eating.
  The age I will give my baby food other than breast milk.
  How often my child sleeps under a bed net.

## DISCUSSION

Understanding the effects of message modality is important when deciding between SMS or voice message services in mHealth programs. Although SMS is a common and effective method, voice messages may be a desirable option based on literacy levels and mobile phone access.

The data presented in this article reveal that delivery success rates for all message modalities are less than ideal. Phone network challenges in rural Balaka were commonly reported by community volunteers and clients throughout the pilot period and likely contributed to the overall low success rates.

Message delivery rates are far more successful among SMS than voice enrollees. Pushing voice messages to clients with personal phones is a complex process, requiring the client to answer the phone at the time of delivery, whereas SMS messages can be delivered at any time, including when the phone is turned off. We believe this is the major reason that SMS messages pushed to personal phones have a higher delivery success rate than voice messages.

During the first few months of implementation, we varied the times that we pushed voice messages to users to find times that clients were most likely to be available to answer the phone. When tips and reminders were launched, voice messages were attempted hourly between 12:00 pm and 6:00 pm local time (CAT) because anecdotal reports indicated that phones were most likely to be charged and switched on during daylight hours. In November 2011, we pushed the voice messages between 2:00 pm and 6:00 pm because we noted more successful attempts occurred during that timeframe. In early 2012, we discovered a configuration problem between the telephone card and the telecom provider that was also contributing to the low delivery success rate. After changing the timing that we pushed the voice messages and fixing the configuration problem, delivery success rates for pushed voice messages increased, but the delivery success rate remained highest for pushed SMS messages.

Retrieved voice messages have the lowest delivery success rate, and this rate decreased over time. Retrieving voice messages from the IVR menu using a community phone requires initiative on behalf of the client who must seek out a volunteer in order to access the community phone. In addition, it requires the volunteer to be available. There were some instances where volunteers moved away from their assigned village or became inactive over time. It is also possible that some community volunteers acted as gatekeepers to the service. We heard anecdotal reports of a small number of volunteers refusing to give out the CCPF telephone number or telling clients that the service must be accessed through a community phone rather than a personal phone.

Furthermore, many community phones broke during the project period, limiting access to the service for community phone users. The phones given to community volunteers were low cost, and approximately 60% of the phones distributed to volunteers were not working 2 years after the original implementation began. It is likely that the unreliability of community phones played a role in the low delivery success of retrieved voice messages in 2 ways:

- Malfunctioning keypads made it difficult to enter an accurate estimated due date or child's birthday in order to hear a message. This is a major reason stated by both volunteers and users for why numerous calls that reached the voice message menu never successfully heard a message.
- Clients who signed up for voice messages in a village where the phone eventually broke were unlikely to find another way to access the service. Some users stated that after the community phone in their village broke, they no longer accessed the CCPF hotline or voice messages.

The data also reveal that delivery success for retrieved voice messages is significantly higher among users subscribed to pregnancy messages than users subscribed to child messages (based on the definition of success used in Table 3). Pregnant users registered an average of 16 weeks before their estimated due date while caregivers of children registered an average of 36 weeks before their child's first birthday. It is possible that the average pregnancy-message enrollee was more likely to take the initiative to visit a community volunteer because the eligibility period was shorter than for the average child-message enrollee. Similarly, it is less likely that a community volunteer would become inactive or the community phone would break during a shorter eligibility period. For pushed message enrollees, there is no clear relationship between message type and success rates because delivery success of voice messages was significantly higher among pregnancy-message enrollees than child-message enrollees, but the opposite was true for pushed SMS messages.

Although delivery success was higher among pushed SMS messages compared with other delivery modalities, the data reveal that clients were highly satisfied with and trusted the voice and SMS messages equally and were likely to learn new information and recall messages regardless of message delivery modality. SMS enrollees, however, were significantly more likely to report intended or actual behavior change than pushed voice enrollees. We suspect that this may be because voice messages can only be heard once while SMS messages can be saved in the client's inbox and read multiple times, and even shared with others. It is also possible that shorter and more frequent messages in the form of SMS are more effective at eliciting intended behavior change than longer voice messages that are delivered once per week. Finally, the differing characteristics of individuals who sign up for SMS messages might make them more willing or able to change their behavior.

Shorter and more frequent messages in the form of SMS may be more effective at prompting behavior change than longer voice messages.

At baseline, this mHealth project discovered that most women in the project area did not own a mobile phone. To address this problem, we provided an additional access point by providing community volunteers with mobile phones. Although the solution was not perfect, our study demonstrates that it is feasible to provide low-literate clients without a personal phone with mobile health information and to stimulate behavior change. Although delivery success was the lowest for retrieved voice message subscribers, approximately 1,200 voice messages were successfully retrieved each month. A similar number of pushed SMS and voice messages were successfully delivered each month. This finding emphasizes the importance of recognizing that mobile health messaging can potentially marginalize populations without access to mobile phones if efforts are not made to adapt mHealth projects to meet the needs of the population. Not surprisingly, phone ownership is inversely correlated with poverty,^21^ and women and people living in rural areas are also less likely to own mobile phones than their male and urban counterparts.^19^ Thus, offering a retrieved voice message option for those without personal phones is an important strategy to deliver health messages to an otherwise hard-to-reach population.

### Limitations

There are a number of important limitations to consider when interpreting the data. First, data from the hotline software (such as demographic data and tips and reminders enrollment status) were entered by hotline workers during calls with clients, and mistakes were common. Implausible data points, such as estimated due dates that were more than 9 months from the call date, had to be excluded. In addition, the data available from the IVR software are by call and cannot be grouped by unique caller, making it difficult to draw conclusions about individual behavior.

Second, our calculation for the number of messages retrieved by voice message subscribers provides only an estimate and is not completely analogous to the success rate for pushed messages. Pushed message success rates are based on 3 attempts while the retrieved message success rate is based on an unknown number of attempts since a subscriber can attempt to retrieve a message an unlimited number of times. Furthermore, some subscribers likely do not try at all. The proportion of message retrieval attempts where a message is successfully played (Figure 2), however, is based on only 1 call attempt and cannot tell us about individual behavior. For example, if 50% of message retrieval attempts are successfully played, we cannot determine whether this translates to 10 unique callers attempting to retrieve a message and only half are successful, or 5 unique callers each trying to retrieve a message twice and failing once and successful on their second try.

There were also a number of limitations to the type of study design used for the phone-based surveys. First, the characteristics of enrollees that largely determined their enrollment type, such as literacy level and access to a personal phone, could be associated with their level of trust, comprehension, and knowledge, and/or behavior change reported during the survey. Thus, any differences observed in survey responses between the different delivery methods may be attributable to characteristics of the sample and not necessarily the delivery method. In addition, among retrieved voice message enrollees, the sample was not randomly selected. Although the community volunteers did not know the purpose of their assignment to identify a client, it is possible that they chose women according to certain characteristics that may bias the sample. We did not collect enough demographic information during the phone surveys to be able to control for potential confounding characteristics. Also, information collected about the enrollees' maternal and child health knowledge and/or behavior change intent relied on self-report, which may present an additional form of bias. Because surveys were conducted by CCPF staff or volunteers, respondents may have wanted to provide answers that they felt were acceptable to the surveyor. Thus, satisfaction, trust, and behavior change indicators may be inflated. On the other hand, respondents were asked to describe their last message and new information learned. Because the information provided was cross-checked against messages sent to the respondent, these indicators are less susceptible to bias. Finally, the sample size is small, making detection of small differences in outcomes difficult.

## CONCLUSION

Mobile technology, specifically SMS and voices messages, can be successfully used to extend health information services to pregnant women and caregivers of young children in rural Malawi. All 3 message modalities led to high levels of satisfaction, comprehension, and new information learned. Due to lower cost, higher delivery success, and higher levels of intent to change behavior, SMS is the preferred delivery modality when possible.

Implementing mHealth projects in areas with low phone penetration creates unique challenges in ensuring access and generating demand. By adapting the service to the context—allowing users to retrieve voice messages from the IVR system without a personal phone and providing community volunteers with phones to serve as access points to the service—this rural population gained access to the mHealth service that would not otherwise have been possible. Although these adaptations presented their own challenges, particularly apparent in the low success rate of retrieved messages, availability of community volunteers, and the difficulty sustaining the volunteer model over time as phones began to break, providing multiple methods by which users could access the service was crucial in extending reach beyond literate personal phone owners.

Further research to understand potential models for increasing access to mobile phones among low-literate, rural populations is warranted. Prior to implementing a similar service, it is important to understand the cost-effectiveness of the intervention and cost implications at scale. At the time of this study, an economic evaluation of CCPF was underway. Finally, further research examining the translation of health information transmitted through mobile phones into healthy behaviors is needed to understand the potential impact of mHealth interventions on their intended health outcomes.
